# Measurement properties of multidimensional patient‐reported outcome measures in neurodisability: a systematic review of evaluation studies

**DOI:** 10.1111/dmcn.12982

**Published:** 2015-12-11

**Authors:** Astrid Janssens, Morwenna Rogers, Rebecca Gumm, Crispin Jenkinson, Alan Tennant, Stuart Logan, Christopher Morris

**Affiliations:** ^1^ PenCRU and PenCLAHRCUniversity of Exeter Medical SchoolUniversity of ExeterExeterUK; ^2^ Royal Devon and Exeter NHS Foundation TrustExeterUK; ^3^ Nuffield Department of Population HealthUniversity of OxfordOxfordUK; ^4^ Department of Rehabilitation MedicineUniversity of LeedsLeedsUK

## Abstract

**Aim:**

To identify and appraise the quality of studies that primarily assessed the measurement properties of English language versions of multidimensional patient‐reported outcome measures (PROMs) when evaluated with children with neurodisability, and to summarize this evidence.

**Method:**

MEDLINE, Embase, PsycINFO, CINAHL, AMED, and the National Health Service Economic Evaluation Database were searched. The methodological quality of the papers was assessed using the COnsensus‐based Standards for selection of health Measurement INstruments checklist. Evidence of content validity, construct validity, internal consistency, test–retest reliability, proxy reliability, responsiveness, and precision was extracted and judged against standardized reference criteria.

**Results:**

We identified 48 studies of mostly fair to good methodological quality: 37 papers for seven generic PROMs (CHIP, CHQ, CQoL, KIDSCREEN, PedsQL, SLSS, and YQOL), seven papers for two chronic–generic PROMs (DISABKIDS and Neuro‐QOL), and four papers for three preference‐based measures (HUI, EQ‐5D‐Y, and CHSCS‐PS).

**Interpretation:**

On the basis of this appraisal, the DISABKIDS appears to have more supportive evidence in samples of children with neurodisability. The overall lack of evidence for responsiveness and measurement error is a concern when using these instruments to measure change, or to interpret the findings of studies in which these PROMs have been used to assess change.

## What this paper adds

- Patient‐reported outcome measures evaluated in children with cerebral palsy, epilepsy, attention‐deficit‐hyperactivity disorder, autism spectrum disorder, and traumatic brain injury.
- Variable methodological quality of studies; improved quality in more recent evaluations.
- Evidence lacking for instruments to assess meaningful changes in health.

This article is commented on by Knox on pages 427–428 of this issue.

**Abbreviations**
ASD: Autism spectrum disorder
COSMIN: COnsensus‐based Standards for selection of health Measurement INstruments
PROM: Patient‐reported outcome measure

Patient‐reported outcome measures (PROMs) assess a patient's health at a single point in time, and are collected through short, self‐completed questionnaires. PROMs are advocated for use in clinical trials;^1^, ^2^ they are also proposed as key performance indicators for evaluating health systems.^3^ Some PROMs are domain‐specific, focusing on a particular aspect of health, such as behaviour; other instruments are multidimensional with sub‐scales that assess various aspects of health and wellbeing. PROMs can be condition‐specific, designed for use by people with a particular health problem; or they can be generic and therefore appropriate for anyone to report their health; or chronic–generic, designed for people with any long‐term health conditions. Preference‐based measures incorporate a weighting of scores based on a reference valuation of health states into a single index score; they are used in economic evaluations to assessing cost‐effectiveness.^2^

‘Neurodisability’ is an umbrella term commonly used in the UK for a range of functional problems of neurological origin. Previously, we proposed a definition of neurodisability for children that was supported by many professionals and parents, and indicated a similar grouping of conditions in other countries, albeit with different terminology.^4^ For some applications in neurodisability, a condition‐specific PROM may be preferable if available; for instance, condition‐specific measures exist for cerebral palsy (CP)^5^ and epilepsy.^6^ However, it is also common for generic PROMs to be utilized in neurodisability, especially for comparison across conditions or with normative samples. Individually, many conditions that result in a neurodisability are rare, but when grouped together they are common. Hence there are situations when it will be expedient for children with neurodisability to be grouped for research, service evaluation, or audits.

When selecting PROMs for a specific purpose, it is necessary to examine both the construct that is being assessed and the measurement properties of candidate instruments.^7^ Mapping of items in PROMs using the International Classification of Functioning, Disability and Health for Children and Youth is useful to understand the content assessed by questionnaires.^8^, ^9^ Measurement properties should, ideally, have been evaluated in samples representative of the intended population to determine whether the instrument is applicable for that population.^1^ Language and cultural issues also affect how people interpret and respond to questions; hence one cannot simply assume that PROMs perform consistently across languages and cultures.^10^, ^11^ Therefore, the US Food and Drug Administration recommends that evidence be provided of the process used to test measurement properties in the language where assessments will be made.^1^

Scale development methodology has evolved in recent years and approaches using item response theory are more commonly utilized; such approaches use mathematical models to examine responses to individual items in questionnaires and offer better scale precision.^12^ Methods for appraising the evidence of psychometric performance on measures have also become more standardized.^13^ The COnsensus‐based Standards for selection of health Measurement INstruments (COSMIN) was developed to enable a standardized assessment of the methodological quality of research studies evaluating measurement properties of tools.^13^, ^14^

In previous papers we documented a systematic review of generic multidimensional PROMs for children and young people, in which we mapped the content to the International Classification of Functioning, Disability and Health for Children and Youth insofar as it was possible,^9^ and appraised studies evaluating measurement properties in general population samples.^15^ In this study we build upon that foundation by focusing on evaluations of the PROMs identified in the previous systematic review where they have been tested in samples of populations with neurodisabilities. In this instance, as the aim was to examine which instruments could be considered robust for application across children with neurodisability, we also included chronic–generic tools. We use the word ‘PROM’ to refer to the group of questionnaires (different versions according to age group, length, or responder) of a certain instrument; we use the word ‘questionnaire’ to refer to a specific version of an instrument. A list of the PROMs, the different types of questionnaires, and full names is presented in Table 1.

**Table 1:** PROMs (group of questionnaires), the different versions (according to age group, length, or responder), and acronyms

Overall PROM name	Acronym questionnaire	Full name questionnaire
CHIP	CHIP‐CE CRF	Child Health And Illness Profile – Child Edition Child Report Form
	CHIP‐CE PRF	Child Health And Illness Profile – Child Edition Parent Report Form (45‐item)
	CHIP‐CE PRF	Child Health And Illness Profile – Child Edition Parent Report Form (76‐item)
CHQ	CHQ‐PF28	Child Health Questionnaire Parent Short Form
	CHQ‐PF50	Child Health Questionnaire Parent Long Form
	CHQ‐CF87	Child Health Questionnaire Child Form (87‐item)
CHSCS‐PS	CHSCS‐PS	Comprehensive Health Status Classification System – Preschool
CQoL	CQoL	Child Quality of Life Questionnaire
DISABKIDS	DISABKIDS DCGM‐37	DISABKIDS Chronic Generic Measure – long form
	DISABKIDS Smileys‐6	DISABKIDS Smiley Measure
EQ‐5D‐Y	EQ‐5D‐Y	EuroQol 5D Youth
HUI	HUI2	Health Utilities Index 2
	HUI3	Health Utilities Index 3
KIDSCREEN	KIDSCREEN‐52	KIDSCREEN‐52
	KIDSCREEN‐10	KIDSCREEN‐10
Neuro‐QOL	Neuro‐QOL	Neurology Quality of Life Measurement System
PedsQL	PedsQL Infant Scales	Pediatric Quality Of Life Inventory Trade Mark 4.0 – Infant Scales
	PedsQL	Pediatric Quality Of Life Inventory Trade Mark 4.0 – Generic Core Scales
	PedsQL SF15 Generic Core Scales	Pediatric Quality Of Life Inventory Trade Mark 4.0 – Short Form 15
SLSS	SLSS	Student Life Satisfaction Scale
	BMSLSS	Brief Multi‐dimensional Student Life Satisfaction Scale
YQoL	YQoL‐S	Youth Quality of Life instrument – Surveillance version
	YQoL‐R	Youth Quality of Life instrument – Research version

## Method

### Search strategy

Candidate generic PROMs were identified and catalogued as part of a previous systematic review.^9^ In addition, chronic–generic PROMs were included as they could be used across neurodisability conditions; three eligible chronic–generic tools were identified in our broader research programme (Disabkids, Functional Disability Index, Neuro‐QoL). For this review, three groups of search terms were combined: the names of the PROMs and their synonyms; terms for children; and terms for neurodisability, for which both free text and medical subject headings were used.

Searches were conducted on MEDLINE (including in‐process and other non‐indexed citations), Embase, PsycINFO, and AMED (via OvidSP), CINAHL (via EBSCOhost), and the National Health Service Economic Evaluation Database (NHS EED; via the Cochrane Library). No date restriction was applied. The searches were run between 12 and 25 September 2012 and updated on 30 July 2014. Forward (checking if key papers had been cited) and backward (checking reference lists) citation chasing was performed for key references to ensure that all relevant literature was retrieved. The electronic search strategy designed for MEDLINE and translated for the other databases is presented in Appendix S1 (online supporting information).

### Inclusion and exclusion criteria

Articles were selected when written in English and reporting on a study that was (1) specifically designed to evaluate the psychometric properties of candidate PROMs using an English language version of the questionnaire, (2) conducted in a population including at least 10% children up to 18 years old with neurodisability, or mixed chronic conditions including neurodisability, and (3) published in a peer‐reviewed journal. Articles were excluded if (1) the PROM was used as a criterion standard to test another instrument, (2) less than 10% of the study population was younger than 18 years, (3) less than 10% of the study sample was diagnosed with neurodisability.

### Study selection

**Figure 1:**
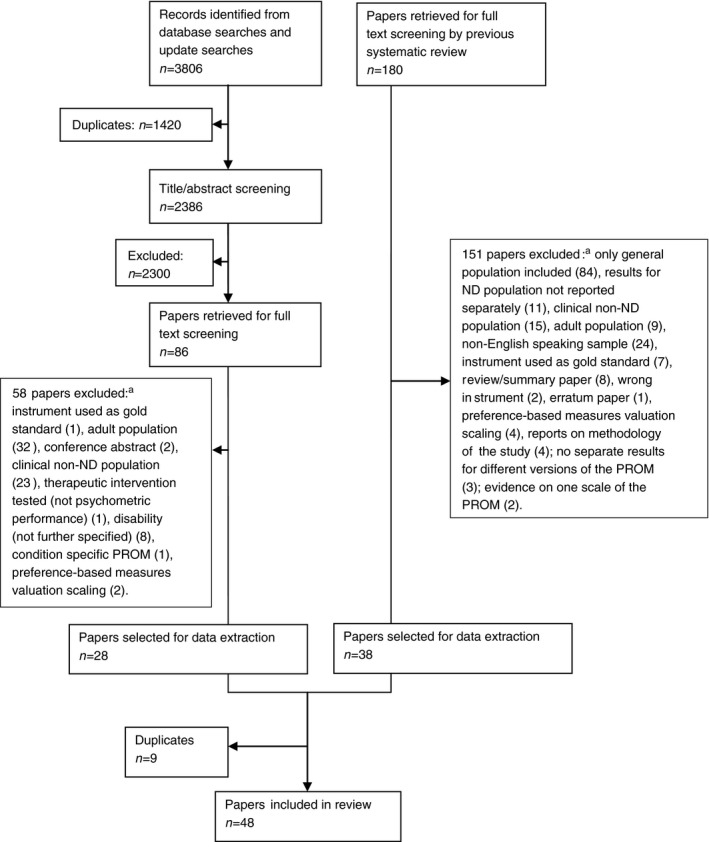
PRISMA flowchart describing identification and selection of studies evaluating psychometric performance of PROMs in a neurodisability population. ^a^Some papers were excluded for more than one reason. PRISMA, Preferred Reporting Items for Systematic Reviews and Meta‐Analyses; PROM, patient‐reported outcome measure.

Titles and abstracts of all unique citations were screened against the eligibility criteria by two reviewers (AJ and RG/CM); any disagreements were resolved by discussion. The full text of any potentially relevant article was retrieved and screened using the same procedure. A flowchart describing the process of study selection can be found in Figure 1.

### Assessment of methodological quality of included articles

For each included paper, the COSMIN checklist was used to appraise the methodological quality of the study and the completeness of the report.^13^ We assessed the methods and reporting of how the following properties had been tested: internal consistency, reliability, measurement error, content validity, structural validity, hypothesis testing, criterion validity, and responsiveness. Cross‐cultural validity was not examined as the purpose of the work was to inform UK health services policy where currently only English language versions are administered. The COSMIN checklist uses a ‘worst score counts’ rating of methodological quality as excellent, good, fair, or poor based on factors such as adequate sample size and appropriate statistical methods used.^16^ The checklist was administered by two reviewers (AJ and CM); discrepancies were resolved by discussion.

### Data extraction

For each included paper the following descriptive data were extracted using a standardized, piloted data extraction form: first author name and year, name and version of the instrument (including child or parent version), study aim, study population (participants’ characteristics including type of neurodisability and diagnosis), number of participants, age range, mean age (and standard deviation), and setting or country where the study was conducted. Data were extracted by one reviewer (RG/AJ) and a 10% sample was checked by a second (AJ/CM).

Then, any data on evidence of the measurement properties of instruments were extracted including content validity (theoretical framework and/or qualitative research), construct validity (structural validity concerning how domain sub‐scales were determined for instance using factor analysis, and hypothesis testing to verify sub‐scales measure the intended construct), internal consistency (including domain sub‐scales where appropriate), test–retest reliability, proxy reliability (between child and parent), precision, and responsiveness (whether the increases/decreases in scores can be considered robust and exceed measurement error). Data were extracted by one reviewer (RG/AJ) and checked by a second (AJ/CM); disagreements were resolved by discussion.

### Appraisal of measurement properties and summary of evidence

Evidence of measurement properties was judged using standardized reference criteria and thresholds (Table 2).^12^, ^17^ These data were summarized in a single rating for each measurement property following methods commonly used for the presentation of such findings.^18^, ^19^ To summarize available evidence, we took into account the following elements (Table 3): (1) data extracted from included studies, with reference to standard criteria (Table 2); (2) the methodological quality of studies (COSMIN) and number of studies; and (3) the thoroughness of testing, giving further weight to any studies that appeared not to have been conducted by the original developers.^20^ Two reviewers (AJ and CM) made the judgement through discussion based on available evidence.

**Table 2:** Appraisal of psychometric properties and indicative criteria

Psychometric property	Indicative criteria
Content validity	Clear conceptual framework consistent with stated purpose of measurement. Qualitative research with potential respondents
Construct validity	Structural validity from factor analysis. Post‐hoc tests of unidimensionality by Rasch analysis. Hypothesis testing, with a priori hypotheses about direction and magnitude of expected effect sizes. Tests for differential item and scale functioning between sex, age groups, and different diagnoses
Reproducibility	Test–retest reliability: intraclass correlation coefficient >0.7 adequate, >0.9 excellent. Proxy‐reliability: child‐ and parent‐reported reliability intraclass correlation coefficient >0.7
Internal consistency	Cronbach's alpha coefficient >0.7 and <0.9
Precision	Assessment of measurement error; floor or ceiling effects <15%; evidence provided by Rasch analysis and/or interval level scaling
Responsiveness	Longitudinal data about change in scores with reference to hypotheses, measurement error, and minimal important difference

**Table 3:** Indices for summarizing appraising psychometric properties of patient‐reported outcome measures

Rating	Definition	
0	Not reported	No studies found that evaluate this measurement property
?	Not clearly determined	Studies were rated poor methodological quality; results not considered robust
−	Evidence not in favour	Studies were rated good or excellent methodological quality; results did not meet standard criteria for this property
+/−	Conflicting evidence	Studies were rated fair, good, or excellent methodological quality; results did not consistently meet standard criteria for this property, e.g. not for all domain scales
+	Some evidence in favour	Studies were rated fair or good methodological quality; standard criteria were met for the property
++	Some good evidence in favour	Studies were rated good or excellent methodological quality; standard criteria were met or exceeded
+++	Good evidence in favour	Studies were rated good or excellent methodological quality; standard criteria were exceeded, results have been replicated

## Results

We found 48 papers that report evaluations of measurement properties of 12 PROMs (see Table 4): 37 papers for seven generic PROMs (CHIP, CHQ, CQoL, KIDSCREEN, PedsQL, SLSS and YQOL), seven papers for two chronic–generic PROMs (DISABKIDS and Neuro QOL), and four papers for three preference‐based measures (HUI, EQ‐5D‐Y and CHSCS‐PS) (Table 4). Twenty papers described evaluations of the PedsQL4.0 in various neurodisability samples, and 11 papers pertained to various versions of the CHQ. The most common conditions in samples were CP, epilepsy, attention‐deficit–hyperactivity disorder (ADHD), autism spectrum disorder (ASD), and traumatic brain injury. The evaluations spanned children in a variety of age groups from 2 to 18 years old. The evaluations were performed in Canada, USA, Europe, and Australia.

**Table 4:** Studies evaluating measurement properties of candidate patient‐reported outcome measures in a population with neurodisability

Instrument version^a^	Reference	Aim/purpose	Study population	*n*	Age range, y	Mean age, y (SD)	Setting, country
CHIP‐CE PR	Riley^35^	To test reliability and validity of the CHIP‐CE with children with ADHD	Children with ADHD in a clinical trial	1476	6–18	Not stated	Outpatient clinics, Europe
CHIP‐CE PR	Schacht^36^	To test reliability and validity of the CHIP‐CE with ADHD	Children with ADHD in five clinical trials	794	6–15	9.7 (2.30)	Outpatient clinics, Europe and Canada
CHQ‐CF87 SR	Landgraf^59^	To test reliability and validity of CHQ‐CF87 with ADHD	General population, subgroup of children with ADHD, and children with end stage renal disease	354 (total) 56 (ADHD) SRFNDP	9–16	11.8 (1.9)	Postal survey, USA
CHQ‐PF28 PR	Pencharz^60^	Evaluate and compare the psychometric properties of the CHQ‐PF‐28 in a paediatric clinical sample	CYP with musculoskeletal disorders, including children with CP and DMD	166 MD: 8 CP: 8	5–16	11.0 (2.9)	Hospital and paediatric rehabilitation centre, Canada
CHQ‐PF50 PR	Vitale^61^	Evaluate and compare the psychometric properties in a paediatric orthopaedic sample	Children with a range of musculoskeletal problems, including CP	242 CP: 23	5–18	12	Physician's office, USA
CHQ‐PF50 PR	Wake^30^	To test reliability and validity of the CHQ‐PF50 with CP	Children with CP	80	5–18	11.25 (3.5)	Outpatient clinics, Australia
CHQ‐PF50 PR	Rentz^29^	To test reliability and validity of the CHQ‐PF50 with children with ADHD	Children with ADHD in a clinical trial	921	6–18	11	Outpatient clinics, USA
CHQ‐PF28 PR	Vitale^62^	To determine the efficacy and sensitivity of the CHQ in children with CP	Children with CP	180	5–18	10.7	Completed before treatment for CP at one hospital, USA
CHQ‐PF50 PR	Thomas‐Stonell^63^	To test responsiveness of the CHQ‐PF50 with TBI	Paediatric patients with TBI	33	4–18	12.5 (4.5)	Inpatient clinic, Canada
CHQ‐PF50 PR	Drotar^64^	To test facture structure of the CHQ‐PF‐50 in a sample of children and adolescents with chronic conditions and physically healthy children seen in a paediatric setting	(1) Children with chronic conditions, including epilepsy (2) General paediatric population	661 (1) 329 (total) Epilepsy: 25 (2) 332	5–18	(1) 12.3 (3.5) (2) 11.4 (3.5)	(1) Outpatient clinics, USA (2) Comparison group from sleep study, USA
CHQ‐PF50 PR	McCullough^31^	To test reliability and validity of CHQ with children with CP	Children with CP	818	8–12	Not stated	Home visits, Europe
CHQ‐PF28 KIDSCREEN‐10 SR and PR	Davis^25^	To compare reliability and validity of the CHQ‐PF28 and Kidscreen‐10	Children with CP	204 (PR) 54 (SR)	4–12	8.25 (2.51)	Outpatient clinics, Australia
CHQ‐PF50 PR	Ferro^65^	To investigate higher‐order factor structure of the CHQ‐PF50 in children with new onset epilepsy	Children newly diagnosed with epilepsy	374	4–12	7.4 (2.3)	Paediatric neurologists’ patients, Canada
CHSCS‐PS PR	Saigal^66^	To develop a multi‐dimensional health status classification system for pre‐school children	(1a) VLBW children and (1b) general population sample (2) VLBW children (3) Children with CP	(1a) 101 (1b) 50 (2) 150 (3) 222 SRFNDP	1–6	(1a) 3.05 (0.09) (1b) 3.04 (0.08) (2) 3.88 (0.62) (3) 3.79 (1.01)	Outpatient clinics, Canada and Australia
CQoL SR and PR	Graham^67^	To develop a QoL measure for 9‐ to 15‐y‐old children, and test it in a healthy and three clinical samples	(1) Children with chronic physical disorders, including neurological disorders; children with mental retardation; children with psychiatric disorders (2) General paediatric population	102 (1) 77 (2) 25	(1) 9–15 (2) 13–14	(1) 12.51–12.97 (1.44–1.79) depending on group (2) 14.03 (0.25)	(1) Outpatient departments and support groups, UK (2) One local school, UK
DCGM‐37 SR	Petersen^68^	To develop and test a chronic–generic HRQoL measure	CYP with different chronic health conditions	360 CP: 21 Epilepsy: 37	6–19	12.48 (2.55)	Outpatient clinics, UK, and six other European countries
DCGM‐37 SR and PR	Schmidt^69^	To test cross‐cultural validity of the DISABKIDS in children with different chronic conditions	Seven CYP groups with different chronic conditions, including CP and epilepsy	122 CP: 27 Epilepsy: 45	8–16	12.12 (2.57)	Seven hospitals, UK, and six other European countries
DCGM‐37 SR and PR	Simeoni^70^	To shorten and test the shortened version of the DISABKIDS in children with chronic diseases	CYP with chronic health conditions, including CP and epilepsy	122 CP: 27 Epilepsy: 45	8–16	12.2 (2.8)	Various clinical settings, UK, and six other European countries
DISABKIDS Smileys‐6 SR and PR	Chaplin^32^	To test the reliability and validity of the DISABKIDS Smiley in children with a chronic disease	CYP with different chronic medical conditions, including CP and epilepsy	435 CP: 56 Epilepsy: 40	4–7	6.04 (1.57)	Hospital clinics, UK, and six other European countries
EQ‐5D‐Y PR	Matza^71^	To test EQ‐5D with children with ADHD, correlations with CHQ‐PF50 and CHIP‐CE	Children with ADHD receiving treatment	126 (total) 83 (UK)	7–18	10.2 (USA) 12.6 (UK) SD not stated	Outpatient clinics, USA and UK
HUI2 SR and PR	Glaser^72^	To assess interrater reliability of the HUI	Children who were CNS tumour survivors	30	6–16	10.5 SD not stated	Outpatient clinics, UK
HUI3 PR	Tilford^73^	To evaluate the construct validity of the HUI‐3 with children with ASD	Children diagnosed with ASD by a multidisciplinary team (DSM‐IV criteria)	150	4–17	8.6 (3.3)	Outpatient clinics, USA
KIDSCREEN‐52 SR and PR	Erhart^33^	To test reliability and validity of KIDSCREEN‐52 in children with CP	Children with CP	828 (total) UK: 144 SRFESP	8–12	10.5 (1.5)	Home visits, Europe
Neuro‐QOL SR and PR	Perez^40^	To identify content area for a health‐related quality of life instrument in neurology	Children with epilepsy and their caregivers	Two focus groups (no numbers stated)	14–20	15.83 (2.23)	Various settings: hospitals, clinics, and patient advocacy associations, USA
Neuro‐QOL SR	Cella^39^	To develop and calibrate the Neuro QOL scales	Children with epilepsy and DMD	59 Epilepsy: 50; MD: 9	Not stated	14.4 (1.9)	Online patient panel and 11 medical centres, USA
Neuro‐QOL SR	Lai^38^	To test reliability and dimensionality of the instrument using computerized adaptive testing	Children with epilepsy and muscular dystrophy	117 Epilepsy: 111 MD: 60	10–21	14.5 (2.8)	Online panel, a medical centre, and clinic, USA
PedsQL 4.0 SR and PR	Eiser^74^	To test inter‐rater reliability (mother or child) and validity	(1) CYP who had survived a CNS tumour (2) CYP with leukaemia	(1) 23 (2) 45 SRFNDP	>8	1) 13.74 (3.06) 2) 13.51 (3.15)	(1) and (2) Recruited at clinic appointment, completed at home, UK
PedsQL 4.0 PR	McCarthy^75^	To test reliability and validity of the PedsQL with TBI	Children and adolescents with TBI or an extremity fracture	391	5–15	10.6 (3.2)	Telephone interviews, USA
PedsQL 4.0 SR and PR	Varni^22^	To test reliability and validity of the PedsQL with children with ADHD	Children with ADHD	72 (SR) 69 (PR)	5–16	10.95 (3.13)	Postal survey, USA
PedsQL 4.0 SR and PR	Varni^76^	To test reliability and validity of the PedsQL with children with CP	Children with CP	77 (SR) 224 (PR)	2–18	8.1 (4.25) (SR) 7.8 (4.0) (PR)	Outpatient clinics, USA
PedsQL 4.0 SR and PR	Varni^21^	To test how young children can self‐report HRQoL using PedsQL	(1) Children with chronic health conditions, including ADHD and CP (2) Healthy children	8591 (SR) 8406 (PR) (1) 2603 (2556 PR) (2) 5988 (5399 PR)	5–16	Not stated	Outpatient clinics and telephone interviews, USA
PedsQL 4.0 SR and PR	Varni^27^	To test the reliability and validity of the PedsQL parent‐proxy report	(1) Children with chronic health conditions, including ADHD and CP (2) Healthy children	(1) 3652 (Total) CP: 250 ADHD: 108 (2) 9467	2–16	Not stated	Outpatient clinics and telephone interviews, USA
PedsQL 4.0 SR and PR	Palmer^77^	To examine the internal consistency and construct validity of the PedsQL brain tumour module and generic core scales	Children with brain tumours	99 (Total) 51 (SR) 99 (PR)	2–18	9.76 (4.52)	Outpatient clinics from one hospital, USA
PedsQL 4.0 SR andPR	Majnemer^78^	To test inter‐rater reliability of PedsQL	Children with CP	48	6–12	9.9 (1.9)	Outpatient clinics, Canada
PedsQL 4.0 SR and PR	Oeffinger^28^	To test longitudinal validity of PedsQL	Children with CP	381	4–18	11 (4.4)	Outpatient clinics, USA
PedsQL 4.0 SR	Varni^79^	To test factorial invariance for the self‐reported PedsQL across different modes of administration	(1) CYP with chronic health conditions, including CP (2) General child and adolescent population	(1) 676 (Total) CP: 70 (2) 1629	5–18	In person: 12.32 (3.59) Mail: 10.24 (3.19) Phone: 11.43 (3.28)	(1) Outpatient clinics and telephone administration, USA (2) Postal survey and telephone administration, USA
PedsQL 4.0 SR	Young^80^	To test the reliability and validity of the web‐based administration of the PedsQL	Children with complex physical health conditions, including CP	69 (Total) CP: 19	8–13	11.0 (1.55)	Clinics in six hospitals/home completion, Canada
PedsQL 4.0 PR	Limbers^81^	To examine the feasibility, reliability, and validity of the PedsQL parent‐proxy in school‐aged children with Asperger syndrome	Children with Asperger syndrome	22	6–12	9.25 (2.15)	Waiting rooms for group social skills class, USA
PedsQL 4.0 SR and PR	Iannaccone^26^	To test reliability and validity of the PedsQL with SMA	Children with SMA	176	2–18	8.53 (4.75)	Outpatient clinics, USA
PedsQL 4.0 SR and PR	Davis^23^	To test reliability and validity of the PedsQL with children with DMD	Children with DMD	44	8–18	12.85 (3.05)	Outpatient clinics, USA
PedsQL 4.0 SR and PR	Dunaway^82^	To test reliability of telephone administration	Children with SMA	20	2–18	8.4 SD not stated	Outpatient clinics, USA
PedsQL 4.0 PR	Limbers^24^	To test reliability and validity of the PedsQL with children with ADHD	Children with ADHD	183	5–18	11.08 (3.7)	Outpatient clinics, USA
PedsQL 4.0 SR	Shipman^83^	To test reliability and validity of the PedsQL with children with ASD	Children with ASD	39	12–18	14.8 SD not stated	Outpatient clinics, USA
PedsQL 4.0 SR and PR	Green^84^	To investigate parent‐adolescent agreement in long‐term QOL outcomes	Adolescents who sustained TBI between birth and 5y old	16	15–18	16.5 (1)	Phone interview, recruited at neurosurgical ward of a children's hospital, Australia
PedsQL 4.0 SR and PR	Sheldrick^85^	To compare adolescent self‐reports with parent reports regarding the QOL of adolescents with ASD	Adolescents diagnosed with ASD at a developmental–behavioural clinic	39	12–18	14.8	Recruited at, and procedures completed at clinic, USA
PedsQL 4.0 SR and PR	Tavernor^86^	To evaluate the content validity of the PedsQL for use with children with ASD	Children diagnosed with ASD	10 (P) 4 (YP)	9–12	Not stated	Recruited via the Database of Children Living with ASDs, UK
SLSS and BMSLSS SR and PR	McDougall^34^	To assess to psychometric properties of the BMSLSS and SLSS in youth with chronic conditions	Adolescents with chronic conditions (including CP, acquired brain injury, and ASD)	439 (Total) CP: 150 (35%) Acquired brain injury: 59 (14%) ASD: 35 (7%)	11–17	Not stated	In a treatment office or adolescent's home, Canada
YQoL‐R SR	Patrick^87^	To develop a quality of life measure for adolescents	Adolescents including samples of general population, ADHD, and mobility disability	236 (Total) 68 (ADHD) 52 (mobility disability)	12–18	Not stated	Outpatient clinics, USA

a Definitions of the instruments are presented in Tables 1 and SI (online supporting information). PR, parent report; ADHD, attention‐deficit–hyperactivity disorder; SR, self‐report; SRFNDP, separate results reported for reported population with neurodisability; CP, cerebral palsy; DMD, Duchenne muscular dystrophy; CYP, Children and young people; SRFESP, Separate results reported for English speaking population; MD, muscular dystrophy; TBI, traumatic brain injury; VLBW, very low birthweight; QoL, quality of life; HRQoL, health‐related quality of life; SMA, spinal muscular atrophy; CNS, central nervous system; ASD, autism spectrum disorder; DSM‐IV, Diagnostic and Statistical Manual of Mental Disorders, Fourth Edition.^88^

The methodological quality of the included studies was variable (Table 5). Internal consistency, test–retest reliability, and construct validity (hypothesis testing) have been more frequently studied in neurodisability samples; several studies have examined structural validity; very few studies have evaluated responsiveness and measurement error. A summary appraisal of the evidence for measurement properties of each PROM is given in Table 6.

**Table 5:** Methodological quality of studies evaluating measurement properties of candidate patient‐reported outcome measures in a population with neurodisability

Instrument version^a^	Author	Internal consistency	Reliability	Measurement error	Content validity	Structural validity	Hypothesis testing	Criterion validity	Responsiveness
CHIP‐CE	Riley^35^	Good				Fair	Good		
CHIP‐CE	Schacht^36^	Fair				Fair	Fair		
CHQ‐CF87	Landgraf^59^	Good					Good		
CHQ‐PF28	Pencharz^60^						Fair		
CHQ‐PF50	Vitale^61^						Fair		
CHQ‐PF50	Wake^30^	Fair					Good		
CHQ‐PF28	Vitale^62^						Fair		
CHQ‐PF50	Rentz^29^	Good		Fair			Good		Good
CHQ‐PF50	Drotar^64^					Poor			
CHQ‐PF50	Thomas‐Stonell^63^								Fair
CHQ‐PF50	McCullough^31^	Excellent				Excellent			
CHQ‐PF28 KIDSCREEN‐10	Davis^25^	Fair					Fair		
CHQ‐PF50	Ferro^65^					Excellent			
CHSCS‐PS	Saigal^66^						Good		
CQoL	Graham^67^	Poor	Poor		Fair				
DCGM‐37	Petersen^68^	Good			Excellent	Good			
DCGM‐37	Schmidt^69^	Good	Good			Good	Good		
DCGM‐37	Simeoni^70^	Good	Good			Good	Good		
DISABKIDS Smileys‐6	Chaplin^32^	Poor	Fair		Excellent		Fair		
EQ‐5D‐Y	Matza^71^						Fair		
HUI2	Glaser^72^		Poor						
HUI3	Tilford^73^						Good		
KIDSCREEN‐52	Erhart^33^					Good	Good		
Neuro QOL	Perez^40^				Poor				
Neuro QOL	Cella^39^ Lai^38^	Poor			Good	Poor			
PedsQL 4.0	Eiser^74^	Poor	Poor						
PedsQL 4.0	McCarthy^75^	Good	Good			Poor	Good		
PedsQL 4.0	Varni^22^	Fair	Fair						
PedsQL 4.0	Varni^76^	Fair	Good				Fair		
PedsQL 4.0	Varni^21^	Fair	Fair				Fair		
PedsQL 4.0	Varni^27^	Fair					Fair		
PedsQL 4.0	Palmer^77^	Poor							
PedsQL 4.0	Majnemer^78^		Fair						
PedsQL 4.0	Oeffinger^28^			Poor					Poor
PedsQL 4.0	Varni^79^					Poor			
PedsQL 4.0	Young^80^		Poor						
PedsQL 4.0	Limbers^81^	Poor					Poor		
PedsQL 4.0	Iannaccone^26^	Fair	Good				Good		
PedsQL 4.0	Davis^25^	Poor	Fair				Fair		
PedsQL 4.0	Dunaway^82^		Poor						
PedsQL 4.0	Limbers^24^	Fair	Fair				Fair		
PedsQL 4.0	Shipman^83^	Poor	Fair				Fair		
PedsQL 4.0	Green^84^		Poor						
PedsQL 4.0	Sheldrick^85^		Fair						
PedsQL 4.0	Tavernor^86^				Fair				
SLSS and BMSLSS	McDougall^34^	Excellent	Excellent			Excellent			
YQoL	Patrick^87^	Fair				Poor	Fair		

a Definitions of the instruments are presented in Tables 1 and SI (online supporting information).

**Table 6:** Summary appraisal of measurement properties in a population with neurodisability

Instrument version^a^	Content validity	Structural validity	Construct validity	Internal consistency	Test–retest reliability	Proxy reliability	Precision	Responsiveness
BMSLSS	0	+	+	+	+	0	0	0
CHIP CE	0	+	+	+	0	0	+	0
CHQ‐CF87	0	+	0	+	0	0	0	0
CHQ‐PF28	0	+	0	−	0	0	−	0
CHQ‐PF50	0	+	+/−	+/−	0	0	−	+/−
CHSCS‐PS	0	0	+	0	0	0	0	0
CQoL	++	0	0	?	?	0	0	0
DCGM‐37	+++	+	++	++	+	−	+	0
DISABKIDS Smileys‐6	++	+	0	+/−	+	0	0	0
EQ‐5D‐Y	0	0	+/−	0	0	0	0	0
HUI2	0	0	0	0	0	?	0	0
HUI3	0	0	+	0	0	0	0	0
KIDSCREEN‐52	0	+	++	0	0	0	+	0
KIDSCREEN‐10	0	0	0	+	+	0	0	0
Neuro‐QOL	+	?	0	?	0	0	0	0
PedsQL 4.0	0	+	?	+/−	++	−	+/−	?
SLSS	0	+	+	+	+	0	0	0
YQoL	0	+	+	+	0	0	0	0

a Definitions of the instruments are presented in Tables 1 and SI (online supporting information).

The PedsQL has been evaluated with children with a wide range of neurodisability including CP, ADHD, ASD, acquired brain injury, neuromuscular, and neuro‐oncology conditions. Although there is supportive evidence for the structural validity and test–retest reliability of the PedsQL, there is conflicting evidence for the internal consistency of the subscales, particularly the school functioning domain, which scored consistently low (0.45–0.65).^21^, ^22^, ^23^, ^24^ Other papers reported values of Cronbach's alpha below 0.7 for emotional functioning,^23^ social functioning,^25^, ^26^ and physical functioning.^22^ We found conflicting evidence for precision; overall floor and ceiling effects were less than 15% for most scales, except social functioning (up to 36%).^21^, ^27^ The responsiveness of the PedsQL was assessed in one poor‐quality study, thus a rating was not determined.^28^ Several studies reported child‐proxy reliability, all reporting low to moderate agreement (intraclass correlation coefficient 0.10–0.75, with most between 0.20 and 0.60).

Versions of the CHQ have been evaluated with children with CP, ADHD, acquired brain injury, and epilepsy. There is supportive evidence for the structural validity of child and parent report versions of the CHQ and internal consistency of the child report version. Both parent versions show poor results for ceiling and floor effects on several scales; ceiling effects are found for most of the individual scales, with scores up to 86% for role and social functioning.^29^ One study reports low values of Cronbach's alpha for the domains family cohesion, bodily pain role/social functioning, and role/social limitations of the 28‐item version;^25^ three studies report conflicting findings for the 50‐item version, with one paper reporting supportive evidence for all domains^30^ and two studies reporting low alpha scores for general health perceptions and family impact (emotional and time).^29^, ^31^ We also found conflicting evidence for the CHQ‐PF50 for construct validity and responsiveness.

The DISABKIDS was developed for and with children who have chronic health conditions including CP and epilepsy. Supportive evidence from methodologically robust studies exists for content validity, construct validity, and internal consistency of the 37‐item version, and there is favourable evidence for structural validity, test–retest reliability, and precision. Evidence did not support child‐proxy reliability. For the 6‐item version for younger children, evidence supports the content validity, structural validity, test–retest reliability, but is conflicting for internal consistency, with values of Cronbach's alpha dropping just below 0.70 for the child version.^32^

Kidscreen‐52 has been evaluated in one study using Rasch analysis with data from children with CP in countries across Europe; the findings for the English language version were reported separately.^33^ Evidence supports structural validity, construct validity, and precision of Kidscreen‐52. Supporting evidence was found for internal consistency and test–retest reliability for the 10‐item version in one study of poorer quality, including children with CP.^25^

One methodologically robust study evaluated the SLSS and BMSLSS with adolescents with conditions including CP, acquired brain injury, and ASD, providing evidence for construct validity, structural validity, internal consistency, and test–retest reliability.^34^

Two papers evaluating the CHIP‐CE parent report version with children with ADHD support structural validity, construct validity, internal consistency and precision.^35^, ^36^

Each of the four preference‐based measures has been evaluated in one study. Evidence from hypothesis testing supports the construct validity of the CHSCS‐PS and HUI3, but was inconsistent for the EQ‐5D‐Y. The child‐proxy reliability of the HUI2 was not rated as the study was of poor quality.

The CQoL was developed with children with intellectual disability, chronic physical disorders, and psychiatric disorders, and parents. The study reporting the development and preliminary testing provides supportive evidence for content validity; internal validity and test–retest reliability could not be determined as these elements of the study were of poor quality.

The YQOL was developed for and with children with disabilities. However, the study reporting on the content validity of the instrument does not state which conditions were included.^37^ A companion paper reports supportive evidence on structural and construct validity, and internal consistency in a study of moderate quality, including children with ADHD or mobility disability.

We identified three papers reporting on the development and initial testing of the Neuro‐QOL; two papers report the same data.^38^, ^39^ Epilepsy and muscular dystrophy were selected as conditions for test development of the paediatric Neuro‐QOL item pool. The content validity, reported in three papers, was rated as good. Domains were identified through a literature review, expert interviews, parent and carer focus groups, and keyword search.^39^, ^40^ Cognitive interviews were conducted with children aged 10 to 18 years to ensure appropriate understanding and literacy levels.^38^ Other measurement properties were not rated owing to the poor quality of the studies.

## Discussion

This review identified 12 multidimensional PROMs, with 18 versions of questionnaires, that have been evaluated with children with various neurodisability conditions, including CP, ADHD, ASD, epilepsy, acquired brain injury, neuromuscular and neuro‐oncology conditions. The PedsQL and CHQ have been evaluated more than other instruments, though some of the evidence undermines confidence in their ability to produce robust measurement. On the basis of this appraisal, the DISABKIDS appears to have more evidence to support its measurement properties in samples of children with neurodisability. None of the PROMs has been evaluated comprehensively across all relevant measurement properties, with responsiveness and measurement error being the least studied.

The paucity of evidence available for the properties of responsiveness and measurement error should be a concern for anyone wishing to use the instruments to measure change, or for those seeking to interpret the findings of studies in which these PROMs have been used to assess change. This gap needs to be evaluated in paediatric populations with neurodisability to inform decisions about what constitutes meaningful change scores. Changes in scores may be statistically significant, especially in large samples, but may not be clinically important. Indices such as the minimal clinically important difference is the mean change in score reported by the respondents who indicate that they had noticed some small change.^41^ The minimal clinically important difference has been evaluated for the PedsQL in a sample of children with diabetes;^42^ nevertheless one cannot necessarily assume this difference will be the same for children with neurodisability conditions. Other ways to address the lack of evidence for responsiveness include the minimum detectable change, which is an indication of the amount of change required to have confidence that any observed change is beyond measurement error; a common standard is to use a 90% confidence level.^43^ The effect size is calculated by dividing the amount of change by the standard deviation of the baseline score.^44^ Revicki et al.^45^ suggest calculating different indices of minimal change, and for these to triangulate towards a range of values, in which confidence increases with replication.

There appears a dearth of evaluations of the measurement properties of preference‐based measures in children with neurodisability, adding to the lack of evidence for these instruments in general populations.^15^ Internal consistency may conflict with the underlying theory of health economic instruments,^46^ but the properties of face, content and construct validity, and test–retest reliability remain requisite. Lack of evidence for these measurement properties undermines confidence in health economic evaluations based on preference‐based measures. We did not examine the methods used to derive the scaling of the preference‐based measures; the methods for creating the preference weighting were assumed to produce interval‐level measurement.^47^ As the purpose of preference‐based measures is to quantify the value or strength of preference for health change, the means for assuming and eliciting preference values should be critically assessed.^46^

The information from this review makes it difficult to recommend a multidimensional PROM for use in paediatric neurodisability based on measurement properties established in relevant conditions. Our review of evaluations of generic multidimensional PROMs in general population samples identified 41 potentially eligible PROMs,^9^ and identified 126 papers that reported evidence of the measurement properties of 25 PROMs using English‐language versions in general population samples.^15^ Although robust evidence was lacking for one or more properties for all PROMs, there was evidence to support more measurement properties for the CHIP, Healthy Pathways,^48^ KIDSCREEN, and MSLSS. The CHU‐9D^49^ was the preference‐based measure with greater evidence of adequate measurement properties. Except for the Healthy Pathways and CHU‐9D, these PROMs have been tested with children and young people affected by neurodisability; the evidence shows a similar pattern albeit supported by fewer studies. Most noticeably absent for all these PROMs are studies examining content validity. Thus these PROMs might be leading candidates for further testing in groups with neurodisability, particularly the properties of responsiveness and longitudinal validity. Tests of responsiveness and longitudinal validity assess how scale scores change over time and whether the direction and magnitude of the changes reflect what would be expected on the basis of theory determined in advance, ideally incorporating a comparison with a group not expected to change. In the absence of evidence of responsiveness, those selecting PROMs should appraise whether the aspects of health assessed by tools and the response options to questions suggest that these are ‘likely’ to change in their specific context for application.

Although PROMs are generally designed for use as group measures in service evaluations, audits, and research, there is also growing interest in using them clinically as individualized measures.^50^, ^51^ The proposed criterion for test–retest reliability is more stringent for individualized use (intraclass correlation coefficient >0.9),^17^ and such high levels of stability would need to be demonstrated in paediatric neurodisability.

Aside from the standard measurement properties, there are several other criteria that apply when selecting a candidate PROM. These include appropriateness, acceptability to potential respondents, and feasibility: for example the burden on respondents and those administering and processing data.^17^ We studied the appropriateness of existing generic and chronic–generic PROMs for children with neurodisability by asking whether they cover the more important aspects of health for this particular group. We sought to identify a core set of outcomes that could be assessed using PROMs for these children; that is, outcomes beyond mortality and morbidity. To this end we performed qualitative research separately with children and parents,^52^ a Delphi survey with health professionals,^53^ and held a prioritization meeting.^54^ This work produced a core set of outcomes deemed important to children and/or parents that were aspects of health targeted by National Health Service clinicians. The domains were communication, emotional wellbeing, pain, sleep, mobility, self‐care, independence, mental health, community and social life, behaviour, toileting, and safety. However, none of the identified PROMs capture all these key domains. Adding to this the scarce evidence of good overall psychometric performance for existing measures in a population with neurodisability, there could be a place to refine or develop existing PROMs accordingly.

There are some limitations to this systematic review; most are a consequence of the strict inclusion criteria. Neurodisability comprises a vast number of conditions, and although we included other general descriptions and MeSH (Medical Subject Headings) terms for developmental disabilities, we only had three key marker conditions (CP, autism, and epilepsy) and relevant variations on neuro‐motor, neuropsychiatric, and developmental disabilities. Although we updated searches for evaluation studies up to 30 July 2014, we did not repeat the systematic search to identify any new PROMs after September 2012. Hence, we will not have included any new PROMs published after this date; however, we are not aware of any such PROMs that would meet the eligibility criteria.

One of our inclusion criteria was published peer‐reviewed reports of studies that specifically set out to evaluate measurement properties of PROMs. Hence, we excluded papers that might have presented incidental evidence from studies where PROMs were used in observational or experimental studies. However, information from studies that were not designed specifically to test measurement properties can be misleading. Studies testing responsiveness require testing of some a priori hypothesis in a longitudinal study, whereas evaluative trials typically test interventions of unknown effectiveness. Therefore, for instance, observing no change could be interpreted as either a blunt, non‐responsive measure or an ineffective intervention, and it is not possible to determine which is true.^55^ In addition, we will have omitted any information that may be contained in manuals, if these data have not been published in peer‐reviewed journals. We justify this as peer review provides some level of quality assurance to the evidence being appraised. We included studies with children and young people with chronic conditions, providing the samples included neurodisability. Hence, we did not appraise studies examining PROMs with children with other conditions (e.g. arthritis or asthma).

Limiting the review to studies where an English version of the PROM was administered excluded some PROMs from further analyses. Two PROMs excluded from this review that may warrant further investigation are ITQoL (for infants),^56^ which was developed in the Netherlands and for which an English translation is available but no published studies of this version were found, and the TNO‐AZL (TACQOL, TAPQOL, and TAAQOL).^57^ If studies had been included that used versions of questionnaires in languages other than English, then further evidence would have emerged, for instance regarding the KINDL^58^ and the plethora of translated versions of the more popular instruments such as PedsQL. Nevertheless, psychometric performance cannot be assumed across languages and cultures;^11^ therefore, in our view, limiting the review to evaluations of English‐language versions is a relative strength of it.

There remains much scope for research in evaluating multidimensional PROMs to measure health outcomes in paediatric neurodisability, particularly in testing item invariance across conditions and the responsiveness of PROM scores to quantify meaningful change that is beyond measurement error.

## Supporting information


**Table SI:** PROMs (group of questionnaires), the different versions (according to age group, length, or responder), acronyms, and reference citations, including reference citation.

**Appendix S1:** An example of the search strategy used on Ovid MEDLINE(R), In‐Process & Other Non‐Indexed Citations, and Ovid MEDLINE(R) (1946 to present).

## References

[1] US Food and Drug Administration . Guidance for Industry: Patient‐Reported Outcome Measures: Use in Medical Product Development to Support Labeling Claims. Rockville, MD: Department of Health and Human Services, Food and Drug Administration, Center for Drug Evaluation and Research, 2009.

[2] National Institute for Health and Care Excellence . Guide to the methods of technology appraisal 2013. http://www.nice.org.uk/article/pmg9/resources/non-guidance-guide-to-the-methods-of-technology-appraisal-2013-pdf (accessed 23 September 2014). 27905712

[3] Fitzpatrick R . In: Smith PC, Mossialos E, Papanicolas I, Leatherman S, editors. Performance Measurement for Health System Improvement: Experiences, Challenges and Prospects. Cambridge University Press, 2009: 63–86.

[4] Morris C , Janssens A , Tomlinson R , Williams J , Logan S . Towards a definition of neurodisability: a Delphi survey. Dev Med Child Neurol 2013; 55: 1103–08. 23909744 10.1111/dmcn.12218

[5] Carlon S , Shields N , Yong K , Gilmore R , Sakzewski L , Boyd R . A systematic review of the psychometric properties of quality of life measures for school aged children with cerebral palsy. BMC Pediatr 2010; 10: 81. 21059270 10.1186/1471-2431-10-81 PMC2995480

[6] Sadeghi S , Fayed N , Ronen GM . Patient‐reported outcome measures in pediatric epilepsy: a content analysis using World Health Organization definitions. Epilepsia 2014; 55: 1431–37. 25131769 10.1111/epi.12740

[7] Rosenbaum P . The ABCs of clinical measures. Dev Med Child Neurol 2015; 57: 496. 25952473 10.1111/dmcn.12735

[8] Fayed N , de Camargo OK , Kerr E , et al. Generic patient‐reported outcomes in child health research: a review of conceptual content using World Health Organization definitions. Dev Med Child Neurol 2012; 54: 1085–95. 22913566 10.1111/j.1469-8749.2012.04393.x

[9] Janssens A , Thompson Coon J , Rogers M , et al. A systematic review of generic multidimensional patient‐reported outcome measures for children, part I: descriptive characteristics. Value Health 2015; 18: 315–33. 25773568 10.1016/j.jval.2014.12.006

[10] Wild D , Eremenco S , Mear I , et al. Multinational trials‐recommendations on the translations required, approaches to using the same language in different countries, and the approaches to support pooling the data: the ISPOR Patient‐Reported Outcomes Translation and Linguistic Validation Good Research Practices Task Force report. Value Health 2009; 12: 430–40. 19138309 10.1111/j.1524-4733.2008.00471.x

[11] Wild D , Grove A , Martin M , et al. Principles of good practice for the translation and cultural adaptation process for patient‐reported outcomes (PRO) measures: report of the ISPOR Task Force for Translation and Cultural Adaptation. Value Health 2005; 8: 94–104. 15804318 10.1111/j.1524-4733.2005.04054.x

[12] Streiner DL , Norman GR . Health Measurement Scales: A Practical Guide to their Development and Use. 3rd edn Oxford: Oxford University Press, 2003.

[13] Mokkink LB , Terwee CB , Knol DL , et al. The COSMIN checklist for evaluating the methodological quality of studies on measurement properties: a clarification of its content. BMC Med Res Methodol 2010; 10: 22. 20298572 10.1186/1471-2288-10-22 PMC2848183

[14] Mokkink LB , Terwee CB , Patrick DL , et al. The COSMIN study reached international consensus on taxonomy, terminology, and definitions of measurement properties for health‐related patient‐reported outcomes. J Clin Epidemiol 2010; 63: 737–45. 20494804 10.1016/j.jclinepi.2010.02.006

[15] Janssens A , Rogers M , Thompson Coon J , et al. A systematic review of generic multidimensional patient‐reported outcome measures for children, part II: evaluation of psychometric performance of English‐language versions in a general population. Value Health 2015; 18: 334–45. 25773569 10.1016/j.jval.2015.01.004

[16] Mokkink LB , Terwee CB , Patrick DL , et al. The COSMIN checklist for assessing the methodological quality of studies on measurement properties of health status measurement instruments: an international Delphi study. Qual Life Res 2010; 19: 539–49. 20169472 10.1007/s11136-010-9606-8 PMC2852520

[17] Fitzpatrick R , Davey C , Buxton MJ , Jones DR . Evaluating patient‐based outcome measures for use in clinical trials. Health Technol Assess 1998; 2: i–iv, 1–74. 9812244

[18] Uijen AA , Heinst CW , Schellevis FG , et al. Measurement properties of questionnaires measuring continuity of care: a systematic review. PLoS ONE 2012; 7: e42256. 22860100 10.1371/journal.pone.0042256 PMC3409169

[19] Davies N , Mackintosh A , Gibbons E , Fitzpatrick R . A structured review of patient‐reported outcome measures forwomenwith breast cancer. (Appendix Bii). Patient‐reported Outcome Measurement Group, University of Oxford, Oxford, UK, 2009 http://phi.uhce.ox.ac.uk/ (accessed 10 February 2015).

[20] McDowell I , Newell C . Measuring Health. A Guide to Rating Scales and Questionnaires. New York, NY: Oxford University Press, 1996.

[21] Varni JW , Limbers CA , Burwinkle TM . How young can children reliably and validly self‐report their health‐related quality of life?: an analysis of 8,591 children across age subgroups with the PedsQL™ 4.0 Generic Core Scales. Health Qual Life Outcomes 2007; 5: 1. 17201920 10.1186/1477-7525-5-1 PMC1769360

[22] Varni JW , Burwinkle TM . The PedsQL as a patient‐reported outcome in children and adolescents with attention‐deficit/hyperactivity disorder: a population‐based study. Health Qual Life Outcomes 2006; 4: 26. 16630344 10.1186/1477-7525-4-26 PMC1459106

[23] Davis SE , Hynan LS , Limbers CA , et al. The PedsQL in pediatric patients with Duchenne muscular dystrophy: feasibility, reliability, and validity of the Pediatric Quality of Life Inventory Neuromuscular Module and Generic Core Scales. J Clin Neuromuscul Dis 2010; 11: 97–109. 20215981 10.1097/CND.0b013e3181c5053b

[24] Limbers CA , Ripperger‐Suhler J , Heffer RW , Varni JW . Patient‐reported pediatric quality of life inventory 4.0 generic core scales in pediatric patients with attention‐deficit/hyperactivity disorder and comorbid psychiatric disorders: feasibility, reliability, and validity. Value Health 2011; 14: 521–30. 21315637 10.1016/j.jval.2010.10.031

[25] Davis E , Shelly A , Waters E , Davern M . Measuring the quality of life of children with cerebral palsy: comparing the conceptual differences and psychometric properties of three instruments. Dev Med Child Neurol 2010; 52: 174–80. 19549193 10.1111/j.1469-8749.2009.03382.x

[26] Iannaccone ST , Hynan LS , Morton A , et al. The PedsQL in pediatric patients with spinal muscular atrophy: feasibility, reliability, and validity of the Pediatric Quality of Life Inventory Generic Core Scales and Neuromuscular Module. Neuromuscul Disord 2009; 19: 805–12. 19846309 10.1016/j.nmd.2009.09.009 PMC2796341

[27] Varni JW , Limbers CA , Burwinkle TM . Parent proxy‐report of their children's health‐related quality of life: an analysis of 13,878 parents’ reliability and validity across age subgroups using the PedsQL™ 4.0 Generic Core Scales. Health Qual Life Outcomes 2007; 5: 2. 17201923 10.1186/1477-7525-5-2 PMC1769359

[28] Oeffinger O , Bagley A , Rogers S , et al. Outcome tools used for ambulatory children with cerebral palsy: responsiveness and minimum clinically important differences. Dev Med Child Neurol 2008; 50: 918–25. 19046185 10.1111/j.1469-8749.2008.03150.x PMC2990955

[29] Rentz AM , Matza LS , Secnik K , Swensen A , Revicki DA . Psychometric validation of the child health questionnaire (CHQ) in a sample of children and adolescents with attention‐deficit/hyperactivity disorder. Qual Life Res 2005; 14: 719–34. 16022065 10.1007/s11136-004-0832-9

[30] Wake M , Salmon L , Reddihough D . Health status of Australian children with mild to severe cerebral palsy: cross‐sectional survey using the Child Health Questionnaire. Dev Med Child Neurol 2003; 45: 194–99. 12613777 10.1017/s0012162203000379

[31] McCullough N , Parkes J , White‐Koning M , Beckung E , Colver A . Reliability and validity of the Child Health QuestionnairePF‐50 for European children with cerebral palsy. J Pediatr Psychol 2009; 34: 41–50. 18499739 10.1093/jpepsy/jsn048

[32] Chaplin JE , Koopman HM , Schmidt S . DISABKIDS Smiley Questionnaire: the TAKE 6 assisted health‐related quality of life measure for 4 to 7‐year‐olds. Clin Psychol Psychother 2008; 15: 173–80. 19115438 10.1002/cpp.570

[33] Erhart M , Ravens‐Sieberer U , Dickinson HO , Colver A . European SPARCLE and Kidscreen Groups. Rasch measurement properties of the KIDSCREEN quality of life instrument in children with cerebral palsy and differential item functioning between children with and without cerebral palsy. Value Health 2009; 12: 782–92. 19490565 10.1111/j.1524-4733.2009.00508.x

[34] McDougall J , Wright V , Nichols M , Miller L . Assessing the psychometric properties of both a global and a domain‐specific perceived quality of life measure when used with youth who have chronic conditions. Soc Indic Res 2013; 114: 1243–57. 25484486 10.1007/s11205-012-0200-z PMC4254371

[35] Riley AW , Coghill D , Forrest CB , Lorenzo MJ , Ralston SJ , Spiel G . Validity of the health‐related quality of life assessment in the ADORE study: parent report form of the CHIP‐Child Edition. Eur Child Adolesc Psychiatry 2006; 15(Suppl. 1): I63–71. 17177018 10.1007/s00787-006-1009-6

[36] Schacht A , Escobar R , Wagner T , Wehmeier PM . Psychometric properties of the quality of life scale Child Health and Illness Profile‐Child Edition in a combined analysis of five atomoxetine trials. Atten Defic Hyperact Disord 2011; 3: 335–49. 21986814 10.1007/s12402-011-0066-y PMC3220810

[37] Edwards TC , Huebner CE , Connell FA , Patrick DL . Adolescent quality of life, part I: conceptual and measurement model. J Adolesc 2002; 25: 275–86. 12128038 10.1006/jado.2002.0470

[38] Lai JS , Nowinski C , Victorson D , et al. Quality‐of‐life measures in children with neurological conditions: pediatric Neuro‐QOL. Neurorehabil Neural Repair 2012; 26: 36–47. 21788436 10.1177/1545968311412054 PMC3710728

[39] Cella D , Nowinski C , Peterman A , et al. The neurology quality‐of‐life measurement initiative. Arch Phys Med Rehabil 2011; 92: S28–36. 21958920 10.1016/j.apmr.2011.01.025 PMC3193028

[40] Perez L , Huang J , Jansky L , et al. Using focus groups to inform the Neuro‐QOL measurement tool: exploring patient‐centered, health‐related quality of life concepts across neurological conditions. J Neurosci Nurs 2007; 39: 342–53. 18186419 10.1097/01376517-200712000-00005

[41] Jaeschke R , Singer J , Guyatt GH . Measurement of health status. Ascertaining the minimal clinically important difference. Control Clin Trials 1989; 10: 407–15. 2691207 10.1016/0197-2456(89)90005-6

[42] Hilliard ME , Lawrence JM , Modi AC , et al. Identification of minimal clinically important difference scores of the PedsQL in children, adolescents, and young adults with type 1 and type 2 diabetes. Diabetes Care 2013; 36: 1891–97. 23340884 10.2337/dc12-1708 PMC3687260

[43] Haley SM , Fragala‐Pinkham MA . Interpreting change scores of tests and measures used in physical therapy. Phys Ther 2006; 86: 735–43. 16649896

[44] Cohen J . Statistical Power Analysis for the Behavioural Sciences. Mahwah, NJ: Lawrence Erlbaum Associates, 1988.

[45] Revicki DA , Cella D , Hays RD , Sloan JA , Lenderking WR , Aaronson NK . Responsiveness and minimal important differences for patient reported outcomes. Health Qual Life Outcomes 2006; 4: 70. 17005038 10.1186/1477-7525-4-70 PMC1586195

[46] Brazier J , Deverill M . A checklist for judging preference‐based measures of health related quality of life: learning from psychometrics. Health Econ 1999; 8: 41–51. 10082142 10.1002/(sici)1099-1050(199902)8:1<41::aid-hec395>3.0.co;2-#

[47] Drummond MF , Sculpher MJ , Torrance GW , O'Brien BJ , Stoddart GL . Methods for the Economic Evaluation of Health Care Programmes. 3rd edn Oxford: Oxford University Press, 2006.

[48] Bevans KB , Riley AW , Forrest CB . Development of the healthy pathways child‐report scales. Qual Life Res 2010; 19: 1195–214. 20563886 10.1007/s11136-010-9687-4 PMC2940033

[49] Stevens KJ . Developing a descriptive system for a new preference‐based measure of health‐related quality of life for children. Qual Life Res 2009; 18: 1105–13. 19693703 10.1007/s11136-009-9524-9

[50] Santana MJ , Haverman L , Absolom K , et al. Training clinicians in how to use patient‐reported outcome measures in routine clinical practice. Qual Life Res 2015; 24: 1707–18. 25589231 10.1007/s11136-014-0903-5

[51] Nelson EC , Eftimovska E , Lind C , Hager A , Wasson JH , Lindblad S . Patient reported outcome measures in practice. BMJ 2015; 350: g7818. 25670183 10.1136/bmj.g7818

[52] Allard A , Fellowes A , Shilling V , Janssens A , Beresford B , Morris C . Key health outcomes for children and young people with neurodisability: qualitative research with young people and parents. BMJ Open 2014; 4: e004611. 10.1136/bmjopen-2013-004611 PMC3996811 24747792

[53] Janssens A , Williams J , Tomlinson R , Logan S , Morris C . Health outcomes for children with neurodisability: what do professionals regard as primary targets? Arch Dis Child 2014; 99: 927–32. 24854564 10.1136/archdischild-2013-305803

[54] Morris C , Janssens A , Shilling V , et al. Meaningful health outcomes for paediatric neurodisability: Stakeholder prioritisation and appropriateness of patient reported outcome measures. Health Qual Life Outcomes 2015; 13: 87. 26108625 10.1186/s12955-015-0284-7 PMC4478638

[55] De Vet HC , Terwee C , Mokkink LB , Knol DL . Measurement in Medicine: A Practical Guide. Cambridge: Cambridge University Press, 2011.

[56] Klassen AF , Landgraf JM , Lee SK , et al. Health related quality of life in 3 and 4 year old children and their parents: preliminary findings about a new questionnaire. Health Qual Life Outcomes 2003; 1: 81. 14690543 10.1186/1477-7525-1-81 PMC331419

[57] Fekkes M , Theunissen NCM , Brugman E , et al. Development and psychometric evaluation of the TAPQOL: a health‐related quality of life instrument for 1–5‐year‐old children. Qual Life Res 2000; 9: 961–72. 11284215 10.1023/a:1008981603178

[58] Ravens‐Sieberer U , Bullinger M . Assessing health‐related quality of life in chronically ill children with the German KINDL: first psychometric and content analytical results. Qual Life Res 1998; 7: 399–407. 9691720 10.1023/a:1008853819715

[59] Landgraf JM , Abetz LN . Functional status and well‐being of children representing three cultural groups: initial self‐reports using the CHQ‐CF87. Psychol Health 1997; 12: 839–54.

[60] Pencharz J , Young NL , Owen JL , Wright JG . Comparison of three outcomes instruments in children. J Pediatr Orthop 2001; 21: 425–32. 11433150

[61] Vitale MG , Levy DE , Moskowitz AJ , et al. Capturing quality of life in pediatric orthopaedics: two recent measures compared. J Pediatr Orthop 2001; 21: 629–35. 11521032

[62] Vitale MG , Roye EA , Choe JC , Hyman JE , Lee FY , Roye DP Jr . Assessment of health status in patients with cerebral palsy: what is the role of quality‐of‐life measures? J Pediatr Orthop 2005; 25: 792–97. 16294138 10.1097/01.bpo.0000164870.26632.6b

[63] Thomas‐Stonell N , Johnson P , Rumney P , Wright V , Oddson B . An evaluation of the responsiveness of a comprehensive set of outcome measures for children and adolescents with traumatic brain injuries. Pediatr Rehabil 2006; 9: 14–23. 16352501 10.1080/13638490500050097

[64] Drotar D , Schwartz L , Palermo TM , Burant C . Factor structure of the Child Health Questionnaire‐Parent Form in pediatric populations. J Pediatr Psychol 2006; 31: 127–38. 16467313 10.1093/jpepsy/jsi078

[65] Ferro MA , Landgraf JM , Speechley KN . Factor structure of the Child Health Questionnaire Parent Form‐50 and predictors of health‐related quality of life in children with epilepsy. Qual Life Res 2013; 22: 2201–11. 23334980 10.1007/s11136-013-0351-7

[66] Saigal S , Rosenbaum P , Stoskopf B , et al. Development, reliability and validity of a new measure of overall health for pre‐school children. Qual Life Res 2005; 14: 243–57. 15789958 10.1007/s11136-004-4228-7

[67] Graham P , Stevenson J , Flynn D . A new measure of health‐related quality of life for children: preliminary findings. Psychol Health 1997; 12: 655–65.

[68] Petersen C , Schmidt S , Power M , Bullinger M , Group D . Development and pilot‐testing of a health‐related quality of life chronic generic module for children and adolescents with chronic health conditions: a European perspective. Qual Life Res 2005; 14: 1065–77. 16041902 10.1007/s11136-004-2575-z

[69] Schmidt S , Debensason D , Muhlan H , et al. The DISABKIDS generic quality of life instrument showed cross‐cultural validity. J Clin Epidemiol 2006; 59: 587–98. 16713521 10.1016/j.jclinepi.2005.09.012

[70] Simeoni M‐C , Schmidt S , Muehlan H , Debensason D , Bullinger M , Group D . Field testing of a European quality of life instrument for children and adolescents with chronic conditions: the 37‐item DISABKIDS chronic generic module. Qual Life Res 2007; 16: 881–93. 17404899 10.1007/s11136-007-9188-2

[71] Matza LS , Secnik K , Mannix S , Sallee FR . Parent‐proxy EQ‐5D ratings of children with attention‐deficit hyperactivity disorder in the US and the UK. Pharmacoeconomics 2005; 23: 777–90. 16097840 10.2165/00019053-200523080-00004

[72] Glaser AW , Furlong W , Walker DA , et al. Applicability of the Health Utilities Index to a population of childhood survivors of central nervous system tumours in the U.K. Eur J Cancer 1999; 35: 256–61. 10448268 10.1016/s0959-8049(98)00367-0

[73] Tilford JM , Payakachat N , Kovacs E , et al. Preference‐based health‐related quality‐of‐life outcomes in children with autism spectrum disorders: a comparison of generic instruments. Pharmacoeconomics 2012; 30: 661–79. 22788258 10.2165/11597200-000000000-00000 PMC3423960

[74] Eiser C , Vance YH , Horne B , Glaser A , Galvin H . The value of the PedsQLTM in assessing quality of life in survivors of childhood cancer. Child Care Health Dev 2003; 29: 95–102. 12603354 10.1046/j.1365-2214.2003.00318.x

[75] McCarthy ML , MacKenzie EJ , Durbin DR , et al. The Pediatric Quality of Life Inventory: an evaluation of its reliability and validity for children with traumatic brain injury. Arch Phys Med Rehabil 2005; 86: 1901–09. 16213229 10.1016/j.apmr.2005.03.026

[76] Varni JW , Burwinkle TM , Berrin SJ , et al. The PedsQL in pediatric cerebral palsy: reliability, validity, and sensitivity of the Generic Core Scales and Cerebral Palsy Module. Dev Med Child Neurol 2006; 48: 442–49. 16700934 10.1017/S001216220600096X

[77] Palmer SN , Meeske KA , Katz ER , Burwinkle TM , Varni JW . The PedsQL Brain Tumor Module: initial reliability and validity. Pediatr Blood Cancer 2007; 49: 287–93. 16991131 10.1002/pbc.21026

[78] Majnemer A , Shevell M , Law M , Poulin C , Rosenbaum P . Reliability in the ratings of quality of life between parents and their children of school age with cerebral palsy. Qual Life Res 2008; 17: 1163–71. 18821030 10.1007/s11136-008-9394-6

[79] Varni JW , Limbers CA , Newman DA . Using factor analysis to confirm the validity of children's self‐reported health‐related quality of life across different modes of administration. Clin Trials 2009; 6: 185–95. 19342471 10.1177/1740774509102309

[80] Young NL , Varni JW , Snider L , et al. The Internet is valid and reliable for child‐report: an example using the Activities Scale for Kids (ASK) and the Pediatric Quality of Life Inventory (PedsQL). J Clin Epidemiol 2009; 62: 314–20. 18834710 10.1016/j.jclinepi.2008.06.011

[81] Limbers CA , Heffer RW , Varni JW . Health‐related quality of life and cognitive functioning from the perspective of parents of school‐aged children with Asperger's syndrome utilizing the PedsQL. J Autism Dev Disord 2009; 39: 1529–41. 19526334 10.1007/s10803-009-0777-5

[82] Dunaway S , Montes J , Montgomery M , et al. Reliability of telephone administration of the PedsQL Generic Quality of Life Inventory and Neuromuscular Module in spinal muscular atrophy (SMA). Neuromuscul Disord 2010; 20: 162–65. 20074950 10.1016/j.nmd.2009.12.002

[83] Shipman DL , Sheldrick RC , Perrin EC . Quality of life in adolescents with autism spectrum disorders: reliability and validity of self‐reports. J Dev Behav Pediatr 2011; 32: 85–89. 21187785 10.1097/DBP.0b013e318203e558

[84] Green L , Godfrey C , Soo C , Anderson V , Catroppa C . Agreement between parent‐adolescent ratings on psychosocial outcome and quality‐of‐life following childhood traumatic brain injury. Dev Neurorehabil 2012; 15: 105–13. 22432599 10.3109/17518423.2011.638331

[85] Sheldrick R , Neger EN , Shipman D , Perrin EC . Quality of life of adolescents with autism spectrum disorders: concordance among adolescents’ self‐reports, parents’ reports, and parents’ proxy reports. Qual Life Res 2012; 21: 53–57. 21505880 10.1007/s11136-011-9916-5

[86] Tavernor L , Barron E , Rodgers J , McConachie H . Finding out what matters: validity of quality of life measurement in young people with ASD. Child Care Health Dev 2013; 39: 592–601. 22515401 10.1111/j.1365-2214.2012.01377.x

[87] Patrick DL , Edwards TC , Topolski TD . Adolescent quality of life, part II: initial validation of a new instrument. J Adolesc 2002; 25: 287–300. 12128039 10.1006/jado.2002.0471

[88] American Psychiatric Association . Diagnostic and Statistical Manual of Mental Disorder. 4th edn Washington, DC: American Psychiatric Association, 2000.

