# Supplementary material for: Measurement properties of multidimensional patient‐reported outcome measures in neurodisability: a systematic review of evaluation studies
Source: Dev Med Child Neurol. 2015 Dec 11;58(5):437–51. doi: 10.1111/dmcn.12982 (PMC5031226; doi:10.1111/dmcn.12982)
Supplement: Supplementary file 1 — Table SI: PROMs (group of questionnaires), the different versions (according to age group, length, or responder), acronyms, and reference citations, including reference citation. [file DMCN-58-437-s001.docx]

**Table SI**: PROMs (group of questionnaires), the different versions (according to age group, length, or responder), acronyms, and reference citations.

| **Overall PROM Name** | **Acronym questionnaire** | **Full name questionnaire** | **First author and year** |
| --- | --- | --- | --- |
| CHIP | CHIP-CE CRF | Child Health And Illness Profile - Child Edition Child Report form | Riley 2004^1^ |
|  | CHIP-CE PRF | Child Health And Illness Profile - Child Edition Parent Report Form (45-item) | Riley 2004^2^ |
|  | CHIP-CE PRF | Child Health And Illness Profile - Child Edition Parent-report Form (76-item) | Riley 2004^2^ |
| CHQ | CHQ-PF28 | Child Health Questionnaire Parent Short Form | Kurtin 1994^3^ |
|  | CHQ-PF50 | Child Health Questionnaire Parent Long Form | Landgraf 1998^4^ |
|  | CHQ-CF87 | Child Health Questionnaire Child Form (87-item) | Landgraf 1997^5^ |
| CHSCS-PS | CHSCS-PS | Comprehensive Health Status Classification System - Preschool | Saigal 2005^6^ |
| CQoL | CQoL | Child Quality of Life Questionnaire | Wasson 1994^7^ |
| DISABKIDS | DISABKIDS DCGM-37 | DISABKIDS Chronic Generic Measure – long form | Simeoni 2007^8^ |
|  | DISABKIDS Smileys-6 | DISABKIDS Smiley Measure | Chaplin 2008^9^ |
| EQ-5D-Y | EQ-5D-Y | EuroQol 5D Youth | Wille 2010^10^ |
| HUI | HUI2 | Health Utilities Index 2 | Torrance 1996^11^ |
|  | HUI3 | Health Utilities Index 3 | Feeny 2002^12^ |
| KIDSCREEN | KIDSCREEN-52 | KIDSCREEN-52 | Ravens-Sieberer 2005^13^ |
|  | KIDSCREEN-10 | KIDSCREEN-10 | Ravens-Sieberer 2010^14^ |
| Neuro-QOL | Neuro-QOL | Neurology Quality of Life Measurement System | Lai 2012^15^ |
| PedsQL | PedsQL Infant Scales | Pediatric Quality Of Life Inventory Trade Mark 4.0 - Infant Scales | Varni 2011^16^ |
|  | PedsQL | Pediatric Quality Of Life Inventory Trade Mark 4.0 Generic Core Scales | Varni 1999^17^ |
|  | PedsQL SF15 Generic Core Scales | Pediatric Quality Of Life Inventory Trade Mark 4.0 - Short Form 15 | Chan 2005^18^ |
| SLSS | SLSS | Student Life Satisfaction Scale | Huebner 1991^19^ |
|  | BMSLSS | Brief Multi‐dimensional Student Life Satisfaction Scale | Seligson 2003^20^ |
| YQoL | YQoL-S | Youth Quality of Life instrument-Surveillance version | Edwards 2002^21^ |
|  | YQoL-R | Youth Quality of Life instrument-Research version | Patrick 2002^22^ |

**REFERENCES**

1 Riley AW, Forrest CB, Rebok GW, Starfield B, Green BF, Robertson JA, Friello P. The Child Report Form of the CHIP-Child Edition: reliability and validity. *Med. Care* 2004; **42**: 221-31.

2 Riley AW, Forrest CB, Starfield B, Rebok GW, Robertson JA, Green BF. The Parent Report Form of the CHIP-Child Edition: reliability and validity. *Med. Care* 2004; **42**: 210-20.

3 Kurtin PS, Landgraf JM, Abetz L. Patient-based health status measurements in pediatric dialysis: expanding the assessment of outcome. *Am. J. Kidney Dis.* 1994; **24**: 376-82.

4 Landgraf JM, Maunsell E, Speechley KN, Bullinger M, Campbell S, Abetz L, Ware JE. Canadian-French, German and UK versions of the Child Health Questionnaire: methodology and preliminary item scaling results. *Quality of Life Research* 1998; **7**: 433-45.

5 Landgraf JM, Abetz LN. Functional status and well-being of children representing three cultural groups: Initial self-reports using the CHQ-CF87. *Psychol. Health* 1997; **12**: 839-54.

6 Saigal S, Rosenbaum P, Stoskopf B, Hoult L, Furlong W, Feeny D, Hagan R. Development, reliability and validity of a new measure of overall health for pre-school children. *Quality of Life Research* 2005; **14**: 243-57.

7 Wasson JH, Kairys SW, Nelson EC, Kalishman N, Baribeau P. A short survey for assessing health and social problems of adolescents. Dartmouth Primary Care Cooperative Information Project (The COOP). *J. Fam. Pract.* 1994; **38**: 489-94.

8 Simeoni M-C, Schmidt S, Muehlan H, Debensason D, Bullinger M, Group D. Field testing of a European quality of life instrument for children and adolescents with chronic conditions: the 37-item DISABKIDS Chronic Generic Module. *Quality of Life Research* 2007; **16**: 881-93.

9 Chaplin JE, Koopman HM, Schmidt S. DISABKIDS Smiley Questionnaire: The TAKE 6 assisted health-related quality of life measure for 4 to 7-year-olds. *Clin. Psychol. Psychother.* 2008; **15**: 173-80.

10 Wille N, Badia X, Bonsel G, Burstrom K, Cavrini G, Devlin N, Egmar A-C, Greiner W, Gusi N, Herdman M, Jelsma J, Kind P, Scalone L, Ravens-Sieberer U. Development of the EQ-5D-Y: a child-friendly version of the EQ-5D. *Quality of Life Research* 2010; **19**: 875-86.

11 Torrance GW, Feeny D, Furlong W, Barr RD, Zhang Y, Wang Q. Multiattribute Utility Function for a Comprehensive Health Status Classification System: Health Utilities Index Mark 2. *Med. Care* 1996; **34**: 702-22.

12 Feeny D, Furlong W, Torrance GW, Goldsmith CH, Zhu Z, DePauw S, Denton M, Boyle M. Multiattribute and single-attribute utility functions for the health utilities index mark 3 system. *Med. Care* 2002; **40**: 113-28.

13 Ravens-Sieberer U, Gosch A, Rajmil L, Erhart M, Bruil J, Duer W, Auquier P, Power M, Abel T, Czemy L, Mazur J, Czimbalmos A, Tountas Y, Hagquist C, Kilroe J, Kidscreen Group E. KIDSCREEN-52 quality-of-life measure for children and adolescents. *Expert Rev. Pharmacoecon. Outcomes Res.* 2005; **5**: 353-64.

14 Ravens-Sieberer U, Erhart M, Rajmil L, Herdman M, Auquier P, Bruil J, Power M, Duer W, Abel T, Czemy L, Mazur J, Czimbalmos A, Tountas Y, Hagquist C, Kilroe J, European KG. Reliability, construct and criterion validity of the KIDSCREEN-10 score: a short measure for children and adolescents' well-being and health-related quality of life. *Quality of Life Research* 2010; **19**: 1487-500.

15 Lai JS, Nowinski C, Victorson D, Bode R, Podrabsky T, McKinney N, Straube D, Holmes GL, McDonald CM, Henricson E, Abresch RT, Moy CS, Cella D. Quality-of-life measures in children with neurological conditions: pediatric Neuro-QOL. *Neurorehabil. Neural Repair* 2012; **26**: 36-47.

16 Varni JW, Limbers CA, Neighbors K, Schulz K, Lieu JE, Heffer RW, Tuzinkiewicz K, Mangione-Smith R, Zimmerman JJ, Alonso EM. The PedsQL Infant Scales: feasibility, internal consistency reliability, and validity in healthy and ill infants. *Qual Life Res* 2011; **20**: 45-55.

17 Varni JW, Seid M, Rode CA. The PedsQL: measurement model for the pediatric quality of life inventory. *Med. Care* 1999; **37**: 126-39.

18 Chan KS, Mangione-Smith R, Burwinkle TM, Rosen M, Varni JW. The PedsQL: reliability and validity of the short-form generic core scales and Asthma Module. *Med. Care* 2005; **43**: 256-65.

19 Huebner E. Initial development of the Student's Life Satisfaction Scale. *Sch. Psychol. Int.* 1991; **12**: 231-40.

20 Seligson JL, Huebner E, Valois RF. Preliminary validation of the Brief Multidimensional Students' Life Satisfaction Scale (BMSLSS). *Social Indicators Research* 2003; **61**: 121-45.

21 Edwards TC, Huebner CE, Connell FA, Patrick DL. Adolescent quality of life, Part I: Conceptual and measurement model. *J. Adolesc.* 2002; **25**: 275-86.

22 Patrick DL, Edwards TC, Topolski TD. Adolescent quality of life, Part II: Initial validation of a new instrument. *J. Adolesc.* 2002; **25**: 287-300.
