# Supplementary material for: Measurement properties of multidimensional patient‐reported outcome measures in neurodisability: a systematic review of evaluation studies
Source: Dev Med Child Neurol. 2015 Dec 11;58(5):437–51. doi: 10.1111/dmcn.12982 (PMC5031226; doi:10.1111/dmcn.12982)
Supplement: Supplementary file 2 — Appendix S1: An example of the search strategy used on Ovid MEDLINE(R), In‐Process & Other Non‐Indexed Citations, and Ovid MEDLINE(R) (1946 to present). [file DMCN-58-437-s002.docx]

**Appendix S1:** An example of the search strategy used on Ovid MEDLINE(R), In-Process & Other Non-Indexed Citations, and Ovid MEDLINE(R) (1946 to present)

1 (15D or 16D or 17D or 15 dimensional or 16 dimensional or 17 dimensional).ti,ab.

2 (AQol or "adolescent quality of life instrument" or "adolescent quality of life mark" or AQol*).ti,ab.

3 (Auquei* or "Autoquestionnaire Qualite de Vie Enfant Image").ti,ab.

4 "Self evaluation of the quality of life of infants".ti,ab.

5 "Pictured Child's Quality of Life Self Questionnaire".ti,ab.

6 Auto Questionnaire Enfant Image.ti,ab.

7 (qualin or "infant's quality of life").ti,ab.

8 (OK*ado or OK ado).ti,ab.

9 "adolescent quality of life questionnaire".ti,ab.

10 (sqlp or "subjective quality of life profile questionnaire for parents").ti,ab.

11 (chaq or Child Health Assessment Questionnaire).ti,ab.

12 chasl.ti,ab.

13 "Child's Health Assessed by Self-Ladder".ti,ab.

14 "Child Health Assessment Ladder".ti,ab.

15 "Health Perception Ladder".ti,ab.

16 (CHIP AE or CHIP CE or "Child Health and Illness Profile").ti,ab.

17 (CHQ or child health questionnaire).ti,ab.

18 (chris or Child Health Ratings Inventories).ti,ab.

19 (CHRS or Children's Health Ratings Scale).ti,ab.

20 (CHSCS PS or "Comprehensive health status classification system for preschool children" or CHSCS*PS).ti,ab.

21 (child health utility or CHU 9D or CHU9D or CHU?9D).ti,ab.

22 CLQI.ti,ab.

23 children* life quality index.ti,ab.

24 (coop or cooperative information project).ti,ab.

25 (CQOL or "child quality of life questionnaire").ti,ab.

26 (disabkids* or dcgm*).ti,ab.

27 (TNO AZL or TNO?AZL or ducatqol or dux* or tapqol or taiqol or taaqol or tacqol).ti,ab.

28 (Euroqol or EQ 5D or EQ 5D* or EQ?5D or (EQ vas or EQ?vas)).ti,ab.

29 (Exqol or EHRQOL or exeter health related quality).ti,ab.

30 functional disability inventory.ti,ab.

31 (disab* and FDI).ti,ab.

32 (FSIIR or functional status II).ti,ab.

33 generic health questionnaire.ti,ab.

34 (GHQ or general health questionnaire or generic children* quality or GCQ).ti,ab.

35 (GWBS or general wellbeing scale or general well being scale).ti,ab.

36 "health and life functioning scale".ti,ab.

37 (HALFS and function*).ti,ab.

38 (HAY adj5 (scale* or questionnaire)).ti,ab.

39 ("how are you" adj10 (scale* or questionnaire*)).ti,ab.

40 HPCRS.ti,ab.

41 healthy pathways child report scale*.ti,ab.

42 (HSCS PS or HSCSPS or health status classification system).ti,ab.

43 (HSQ or health status questionnaire).ti,ab.

44 (hui or health utilities index).ti,ab.

45 comprehensive health status classification system.ti,ab.

46 chscs.ti,ab.

47 (IPQ or illness perception* questionnaire).ti,ab.

48 (ITQoL or "infant toddler quality of life scale" or (infant toddler and "quality of life")).ti,ab.

49 (KINDL or KINDLR).ti,ab.

50 nottingham health profile.ti,ab.

51 ("Nordic quality of life questionnaire" or Nordic QOLQ or Nordic quality of life questionnaire).ti,ab.

52 (pediatric healthquiz or pediatric health quiz or paediatric healthquiz).ti,ab.

53 (pedsql or "pediatric quality of life inventory").ti,ab.

54 (PIE scale* or "perceived illness experience").ti,ab.

55 PWI SC.ti,ab.

56 personal wellbeing index.ti,ab.

57 (comqol or "comprehensive quality of life scale").ti,ab.

58 (QLQC or "quality of life questionnaire for children").ti,ab.

59 (QoLAQ or "quality of life assessment questionnaire").ti,ab.

60 (("quality of life profile" and adolescent) or QOLP AV).ti,ab.

61 ("quality of my life questionnaire" or QoML).ti,ab.

62 (quality of well being scale or quality of wellbeing scale or "quality of well being self administered" or "quality of wellbeing self administered" or QWB).ti,ab.

63 (RAND and (health insurance or health survey)).ti,ab.

64 (RAND HIS or RAND HSMC).ti,ab.

65 (CHSQ or child health status questionnaire).ti,ab.

66 (SF and health survey).ti,ab.

67 (MOS SF* or short form health survey).ti,ab.

68 (sickness impact profile or SIP questionnaire).ti,ab.

69 (student* life satisfaction scale or slss or mslss).ti,ab.

70 (wchmp or (warwick child health and morbidity profile)).ti,ab.

71 (whoqol or "world health organisation quality of life").ti,ab.

72 (yoq or youth outcome questionnaire).ti,ab.

73 (YQOL or "youth quality of life instrument*").ti,ab.

74 or/1-73

75 child/

76 child*.ti,ab.

77 adolescent/

78 adolescent*.ti,ab.

79 infant/

80 infant*.ti,ab.

81 75 or 76 or 77 or 78 or 79 or 80

82 exp Nervous System Diseases/

83 Neurologic Manifestations/

84 Autistic Disorder/

85 developmental disabilities/ or learning disorders/ or intellectual disability/ or motor skills disorders/

86 exp cerebral palsy/

87 (cerebral adj palsy).ti,ab.

88 epilep*.ti,ab.

89 autis*.ti,ab.

90 (neuro-motor adj disease*).ti,ab.

91 (neuromotor adj disease*).ti,ab.

92 (neuromotor adj disorder*).ti,ab.

93 (neuro-motor adj disorder*).ti,ab.

94 (neuromotor adj dysfunction*).ti,ab.

95 (neuro-motor adj dysfunction*).ti,ab.

96 neurodisabilit*.ti,ab.

97 (neuropsychiatric adj disease*).ti,ab.

98 (neuropsychiatric adj dysfunction*).ti,ab.

99 neuro-psychiatric.ti,ab.

100 ((Child* or infant* or adolescen*) adj4 disab*).ti,ab.

101 or/82-100

102 74 and 81 and 101

103 (duke health profile or dhp-a).ti,ab.

104 81 and 101 and 103

105 102 or 104
